# SUPPORTING AGING IN PLACE THROUGH IWISH: IMPACTS FROM THE SUPPORTIVE SERVICES DEMONSTRATION

**DOI:** 10.1093/geroni/igae098.0021

**Published:** 2024-12-31

**Authors:** Ian Breunig, Melissa Vandawalker, Kimberly Groover, Derek Hoodin

**Affiliations:** Abt Global, Nashville, Tennessee, United States; Abt Global, Cambridge, Massachusetts, United States; Abt Global, Cambridge, Massachusetts, United States; Abt Global, Cambridge, Massachusetts, United States

## Abstract

The U.S. Congress sponsored the Supportive Services Demonstration (SSD) to learn whether structured health and wellness support can help older adults remain in HUD-assisted housing developments longer. The demonstration tested the Integrated Wellness in Supportive Housing (IWISH) model, which funds both an onsite Resident Wellness Director and part-time Wellness Nurse at multifamily properties to address health, housing, and social service needs of adults ages 62 and older. Through a cluster-randomized controlled trial including properties in seven states, SSD tests whether IWISH affects: (1) the length of stay in housing by reducing transitions to long-term care, (2) unplanned hospitalizations and other acute healthcare utilization, and (3) primary care utilization. We linked individual resident data with Medicare and Medicaid data and compared utilization and tenure between IWISH and control residents. During the first three years of the demonstration (October 2017–September 2020), we found no evidence that IWISH affected residents’ healthcare utilization or caused residents to remain in their homes longer; but there are reasons to be optimistic about the longer-term impact of the IWISH model. Subgroup analyses show IWISH reduced rates that residents aged 62-64 and residents 85 or older used acute care services. We also found two aspects of the IWISH model seemed to improve outcomes: working individually with residents on meeting their health and wellness goals and providing transitional care to residents returning home from a hospital or long-term care stay. Outcomes were worse at properties that are isolated or lacking access to nutritional food.

